# Diagnostic heterogeneity in psychiatry: towards an empirical solution

**DOI:** 10.1186/1741-7015-11-201

**Published:** 2013-09-12

**Authors:** Klaas J Wardenaar, Peter de Jonge

**Affiliations:** 1 Interdisciplinary Center Psychopathology and Emotion Regulation (ICPE), University of Groningen, University Medical Center Groningen (UMCG), Hanzeplein 1, Groningen, 9713 GZ, The Netherlands

**Keywords:** DSM-5, Heterogeneity, Data-driven techniques, Cattell’s cube

## Abstract

The launch of the 5th version of the Diagnostic and Statistical Manual of Mental Disorders (DSM-5) has sparked a debate about the current approach to psychiatric classification. The most basic and enduring problem of the DSM is that its classifications are heterogeneous clinical descriptions rather than valid diagnoses, which hampers scientific progress. Therefore, more homogeneous evidence-based diagnostic entities should be developed. To this end, data-driven techniques, such as latent class- and factor analyses, have already been widely applied. However, these techniques are insufficient to account for all relevant levels of heterogeneity, among real-life individuals. There is heterogeneity across persons (p*:*for example, subgroups), across symptoms (s*:*for example, symptom dimensions) and over time (t*:*for example, course-trajectories) and these cannot be regarded separately. Psychiatry should upgrade to techniques that can analyze multi-mode (p-by-s-by-t) data and can incorporate all of these levels at the same time to identify optimal homogeneous subgroups (for example, groups with similar profiles/connectivity of symptomatology and similar course). For these purposes, Multimode Principal Component Analysis and (Mixture)-Graphical Modeling may be promising techniques.

## Introduction

With the launch of the fifth version of the Diagnostic and Statistical Manual of Mental Disorders (DSM-5), the debate about current psychiatric diagnostics has come into the limelight again, focusing on specific alterations in the DSM-5, such as the deletion of pervasive developmental disorder not otherwise specified (PDD-NOS) and Asperger’s Disorder [1,2] and the inclusion of mourning in major depressive disorder (MDD). However, more fundamental topics,such as the medicalization of normal behavior [3] and the categorical approach to continuous phenomena, are also debated [4]. Perhaps the most important criticism of the DSM-5 regards the poor validity of its classification. Several researchers have even stressed that the DSM-5 hampers research into the underlying mechanisms in the etiology of psychopathology and that the current state of affairs is one of scientific stagnation [5]. We argue that the development of more valid psychiatric classifications is important in order to link mental states to specific causes in scientific research, and that this process should be evidence-based. Decreasing the amount of diagnostic heterogeneity is central in this process.

### The problem of diagnostic heterogeneity

Current psychopathological concepts are heterogeneous by default whichrestricts their usefulness for research [6,7]. In the past, evidence-based attempts to decrease heterogeneity have been made. For depression, for instance, subtypes have been identified with latent class analyses (LCA) [8,9], symptom-dimensions with factor analyses (FA) [10,11] and course-trajectory groups with mixture growth analyses (MGA) [12,13]. Unfortunately, these studies tackle only one aspect of heterogeneity at a time. LCA focuses on person (p)-level heterogeneity, but does not account for within-class symptom and course variations. FA tackles symptom (s)-level heterogeneity, but assumes stability across persons and time. MGA describes temporal (t) heterogeneity, but does not account for s-level heterogeneity. Not surprisingly, these approaches have led to artificial models with limited replicability [11].

### The solution: simultaneous heterogeneity reduction

**Figure 1:**
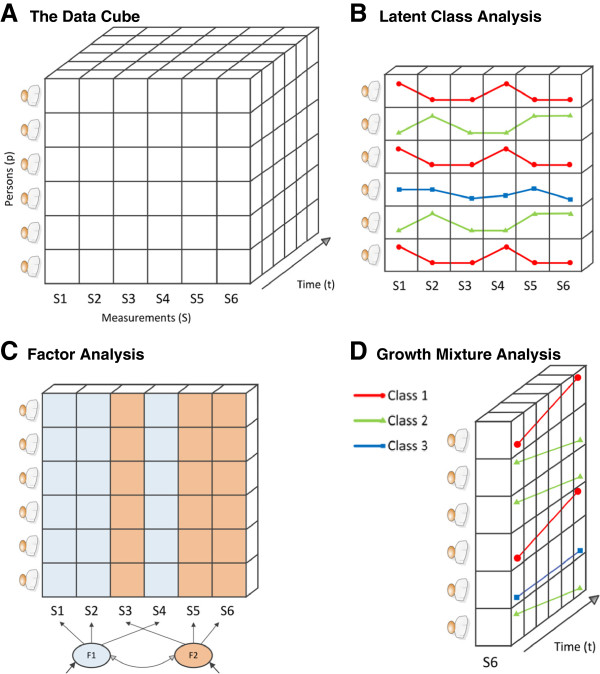
Cattell’s ‘data-cube’ (A), latent class analysis with three classes (red, green, blue) in the S-by-P slice (B), factor analysis with two factors within the S-by-P slice (C) growth mixtureanalysis with three classes (red, green, blue) within the P-by-T slice (D).

If homogeneous diagnoses are what psychiatry aims for, a data-driven approach should be designed to minimize heterogeneity on each level simultaneously. To enable reduction of p-, s- and t-level heterogeneity, three-mode data are needed, visualized by Cattell’s data cube [14] (Figure 1A). The cube consists of measured data (s-axis) for n individuals (p-axis) at k time-points (t-axis). For each combination of axes (slices), different statistical techniques apply. Cross-sectional studies of heterogeneity apply to the p-by-s slice: LCA divides the p-axis into classes (Figure 1B) and FA divides the s-axis into factors (Figure 1C). To model heterogeneity of the whole slice, model combinations (for example,factor mixture models) [15] can be used. Longitudinal studies of heterogeneity (for example, MGA) apply to the p-by-t slice, modeling classes-based temporal trajectories on one or more variables (Figure 1D). Although incomplete, this summary shows that none of these models incorporate all three sources of variation. If we look to other fields (for example, psychometrics, mathematics), we can see that statistical advances have reached the point where ‘three-dimensional models’ are a possibility. Here, we briefly discuss two candidates.

### The latent variable approach: three-mode principal component analysis (3MPCA)

3MPCA [16] is an exploratory technique, designed to decompose the latent structure of three-dimensional data by identifying the number of components that make up each of the axes. Investigation of the interactions between the modes can yield insights into the latent structure of three-dimensional data as a whole. In anxiety patients, for instance, 3MPCA showed that patients could be divided into subgroups (p-component) with different clusters of symptoms (s-component) in different situations (t-component) [17]. Such an approach can be extended to a broader range of psychopathological phenomena. 3MPCA does have its limitations: it requires subjective judgments to enable modelselection and can yield hard-to-interpret results. However, it is a fully developed technique that can be used to explore three-dimensional psychopathology data for more homogeneous diagnostic entities.

### The network approach: (mixture) graphical analysis

Traditional concepts of psychopathology (diagnoses, subtypes, dimensions) lean heavily on the assumption that corresponding latent constructs exist. Unfortunately, it is uncertain to what extent this is a realistic assumption [18]. Rather than assuming that different symptoms (energy loss, suicidal ideation) are caused by one underlying disease (for example, depression), one could instead look at how symptoms interact, amplify and sustain each other over time in a network of symptoms (nodes) and causal links (edges) [18,19], using graphical model methodology, developed in biostatistics [20]. Such patient-descriptions are highly personalized: they take homogeneity to the extreme, both at the s- and p-level. Within three-dimensionaldata the s-axis is completely subdivided down to its smallest components (for example, symptoms). On the p-axis, for each person, the repeatedly measured symptoms are incorporated in a personalized network model. On the p-level, such an approach could lead to an indefinite number of possible network configurations, leaving us without any common denominators. However, subgroups with common network characteristics can be identified by mixture/latent class analyses on networkmodel parameters. Such an approach can yield subtypes that are not merely defined by common symptomatology, but particularly by their observed interconnectedness.

## Conclusions

The development of evidence-based diagnoses in psychiatry is bound to require the use of datadriven techniques. In order for the resulting diagnostic models to optimally reflect real-world variation among patients, multiple sources of heterogeneity should be simultaneously evaluated. Although complex, and dependent upon the dataquality, such methods are a necessity when psychiatric diagnosis seeks an empirical basis.

## Competing interests

The authors declare that they have no competing interests.

## Pre-publication history

The pre-publication history for this paper can be accessed here:

http://www.biomedcentral.com/1741-7015/11/201/prepub
